# Eye Tracking the Face in the Crowd Task: Why Are Angry Faces Found More Quickly?

**DOI:** 10.1371/journal.pone.0093914

**Published:** 2014-04-03

**Authors:** Jonathon R. Shasteen, Noah J. Sasson, Amy E. Pinkham

**Affiliations:** 1 School of Behavioral and Brain Sciences, The University of Texas at Dallas, Dallas, Texas, United States of America; 2 Department of Psychology, Southern Methodist University, Dallas, Texas, United States of America; 3 Department of Psychiatry, The University of Texas Southwestern Medical School, Dallas, Texas, United States of America; Tel Aviv University, Israel

## Abstract

Among a crowd of distractor faces, threatening or angry target faces are identified more quickly and accurately than are nonthreatening or happy target faces, a finding known as the “face in the crowd effect.” Two perceptual explanations of the effect have been proposed: (1) the “target orienting” hypothesis (i.e., threatening targets orient attention more quickly than do nonthreatening targets and (2) the “distractor processing” hypothesis (i.e., nonthreatening distractors paired with a threatening target are processed more efficiently than vice versa, leading to quicker detection of threatening targets). Using a task, with real faces and multiple identities, the current study replicated the face in the crowd effect and then, via eye tracking, found greater support for the target orienting hypothesis. Across both the classical search asymmetry paradigm (i.e., one happy target in a crowd of angry distractors vs. one angry target in a crowd of happy distractors) and the constant distractor paradigm (i.e., one happy target in a crowd of neutral distractors vs. one angry target in a crowd of neutral distractors), fewer distractors were fixated before first fixating angry targets relative to happy targets, with no difference in the processing efficiency of distractors. These results suggest that the face in the crowd effect on this task is supported to a greater degree by attentional patterns associated with properties of target rather those of the crowd.

## Introduction

Referred to as the “face in the crowd effect,” angry or threatening target faces are identified more quickly and accurately, among a crowd of distractor faces, than are nonthreatening or happy faces [1] [2] [3] [4]. This advantage for detecting social threat is typically examined using paradigms in which several facial expressions are presented simultaneously. In one common version of such tasks, participants are asked to decide whether all of the displayed faces express the same emotion or one of the faces expresses a discrepant emotion. In both the classical search asymmetry paradigm (i.e., one happy target in a crowd of angry distractors vs. one angry target in a crowd of happy distractors) and the constant distractor paradigm (i.e., one happy target in a crowd of neutral distractors vs. one angry target in a crowd of neutral distractors), response times (RTs) for detecting the discrepant target face are compared across conditions in order to determine if one emotion type is detected more quickly than is another [1]. The face in the crowd effect is supported if an angry face in a crowd of distractor faces is found more quickly and/or accurately than is a happy face in a crowd of distractor faces.

Studies that utilize schematic faces (i.e., line drawings) as stimuli typically reveal an advantage for detecting angry faces [2] [5] [6], leading some to argue that the face in the crowd effect reflects an evolutionary advantage for quickly detecting environmental threat, based on affective properties [1] [2]. However, schematic faces have been criticized for maximizing perceptual control at the expense of ecological validity [1] [7]. Although studies using veridical faces as stimuli may better approximate the effect as a real-world phenomenon, they have produced mixed results. Some show an advantage for angry expressions [1] [3] [4], but others demonstrate either no difference or an advantage for happy expressions [7] [8] [9] [10] [11]. In addition, after controlling for bottom-up visual saliency with realistic human faces, Ceccarini and Caudek [12] found strong support for an anger advantage when using dynamic stimuli but not when using static images. These inconsistencies have generally been explained by emphasizing either perceptual [11] or emotional factors [10] [13]. Those emphasizing perceptual properties of emotional faces, rather than a selective advantage for threat-specific detection, have been supported by studies showing that manipulating perceptual confounds, such as the amount of teeth displayed, can influence the speed and accuracy with which target faces are detected [10] [14] [15]. In contrast, emotional explanations argue that affective cues drive superior detection, and have been support most recently by a reanalysis of prior findings suggesting that emotional arousal may explain previous inconsistencies in the literature [16]. Although these debates are ongoing, most agree that methodological variation across tasks contributes to discrepancies in prior findings.

Recently, Pinkham and colleagues [3] developed a new version of the Face in the Crowd Task to determine if the more robust effects generated using schematic faces could be replicated with ecologically valid stimuli. Here, photographs of happy, angry and neutral faces were selected for inclusion after validation using the Facial Action Coding System [17]. Further, unlike schematic designs that often construct crowds using identical faces, no individual in this task is presented twice within the same matrix, resulting in a more “true” crowd effect. The face in the crowd effect was found using this task across both the constant distractor and search asymmetry paradigms [3]. Subsequent studies have replicated these findings in other typically developing adult populations [18] [19], while also reporting impaired performance in schizophrenia on the task [18], as well as enhanced performance by riot police trained to rapidly identify signals of threat [19].

The attentional processes underlying superior detection of angry faces on this task, however, remain unclear. More generally, the face in the crowd effect has been hypothesized to arise from two distinct but not mutually exclusive attentional processes: the “target orienting” hypothesis and the “distractor processing” hypothesis. The “target orienting” hypothesis argues that, due to enhanced salience, angry target faces orient attention more quickly than do happy target faces [5]. Supporting this hypothesis, eye tracking of schematic faces has demonstrated that fewer fixations occur before the first fixation on negative targets than on positive targets [20]. Moreover, in this study, the first saccade (i.e., a rapid shift of the eye between fixation points) was more likely to orient attention to negative targets than to positive targets. Thus, increased initial saccade accuracy and fewer fixations before first target fixation are congruent with theories that suggest that targets can increase preattentive guidance, irrespective of distractor type [5] [21]. Behaviorally, the constant distractor paradigm also offers support for the target orienting hypothesis as it has elicited higher accuracy rates and faster RTs for angry targets than for happy targets within identical neutral crowds [3] [5]. These findings have led many to infer that affective and/or perceptual properties of angry *targets*, rather than the differential processing of distinct distractor types found in the search asymmetry paradigm, underlie the face in the crowd effect.

Whether the target orienting hypothesis is supported within the search asymmetry paradigm is even less clear, as the face in the crowd effect found in this paradigm has frequently been explained as an effect of the distractor rather than the target. This “distractor processing” hypothesis contends that the effect on the search asymmetry paradigm is influenced at least in part by differential processing of distractors, such that happy distractors are processed more efficiently than are angry distractors [6]. In other words, angry distractors hold attention longer than do happy distractors; thus, quicker detection of angry targets is a by-product of faster search through happy distractors, whereas slower detection of happy targets is due to relatively slower search through angry distractors. Supporting this view, prior research has shown that happy expressions presented individually are categorized more accurately [22] [23] and more quickly [26] than are angry expressions. Similarly, individuals visually disengage more quickly from happy faces than from angry faces, when both are presented simultaneously [25]. Also suggesting an advantage for the efficient processing of happy faces, other studies have shown that placing the target further away from the center of the matrix slows RT [26] [27] and that increasing the amount of distractor faces slows the detection of positive target faces more so than of negative target faces [5] [20] [21]. Considered together, these results demonstrate that target detection may be influenced in part by the speed of processing distractors, which may also differ depending on the emotional category of the crowd.

Hence, prior studies reveal evidence supporting both the “target orienting” and “distractor processing” hypotheses. Because this evidence likely reflects methodological differences between tasks, including but not limited to the use of search asymmetry and/or constant distractor paradigms, here we directly evaluate the relative contribution of each hypotheses using eye tracking on a common task that includes both paradigms, the Face in the Crowd task [3]. This task was selected for the current study not only for its high ecological validity, but also because it is particularly well-suited for analysis via eye tracking. Unlike traditional visual search paradigms, the Face in the Crowd task does not include any manipulation of set size. Although such manipulation can confer several benefits when analysis focuses exclusively on behavioral data, it also increases subject burden and can complicate interpretation, particularly for eye tracking data. Thus, the Face in the Crowd task was selected because it provides a parsimonious design for examining visual attentional patterns underlying superior detection of angry targets within both the constant distractor and search asymmetry paradigms.

In the present study, eye tracking indices used in previous work [20] were selected to evaluate both the target orienting and distractor processing hypotheses on the Face in the Crowd task. Although previous studies have examined the target orienting and distractor processing hypotheses by inferring support based upon RT and accuracy, to our knowledge this study is the first to explicitly assess both hypotheses using eye tracking on a common task consisting of real faces. Fixation and eye movement analysis has been shown to be a valid indicator of visual attention [28] that is sensitive to differentiating search strategies [29] [30]. The predicted behavioral effects of faster and more accurate detection of angry targets were anticipated to coincide with shorter latencies to first fixate angry faces, after matrix onset, compared to happy faces. Such a difference in latency by target type, however, could be explained by greater attention-orienting to threatening targets and/or by more efficient processing of nonthreatening distractors. To support the target orienting hypothesis, eye tracking analysis should reveal that fewer distractors are fixated when the target is threatening, compared to nonthreatening. Fixating fewer members of the crowd prior to finding angry targets, when compared to happy targets, would suggest that angry targets are more quickly identified at least in part because they disproportionately orient attention. To support the distractor processing hypothesis, the average duration of fixation time per distractor viewed, prior to fixating the target, should be shorter for nonthreatening distractors paired with a threatening target than vice versa. Such evidence would suggest that angry targets are found more quickly than are happy targets at least in part because nonthreatening crowds are processed more efficiently than are threatening ones. Because these hypotheses are not necessarily mutually exclusive, it is possible that evidence supporting both may be found. Such a finding would indicate that superior detection of angry faces within the task used here is supported by both properties of the target and those of the distractors.

Additionally, although assessment of both hypotheses focuses on explaining target effects, the task used here also enables the examination of previously reported distractor effects of emotional relative to neutral crowds. For example, prior face in the crowd studies have reported that angry crowds slow detection of happy targets more so than neutral crowds [3] [6] [7] [19]. Such a comparison of emotional versus neutral distractors will also be examined, as it addresses a distinct question from the distractor processing hypothesis, which focuses on the more primary goal of assessing potential processing differences between emotional distractor types (i.e., angry vs. happy distractors).

## Methods

### Ethics Statement

All participants provided written informed consent prior to study participation. The protocol for this study was approved by The University of Texas at Dallas Institutional Review Board.

### Participants

Thirty-three undergraduate students (15 male) from The University of Texas at Dallas participated, ranging in age from 19 to 46 years with a mean of 24.4 (*SD* = 7.38) years. Each received course credit in exchange for participating.

### Stimuli and Apparatus

The face in the crowd effect was examined using the Face in the Crowd Task [3]. Stimuli were twenty-seven photographs of nine individuals showing angry, happy and neutral facial expressions chosen from a larger database of facial displays of emotion by individuals who had consented to having their images included in tasks and publications [31]. All expressions included in the task were well-validated using both objective ratings, based on the Facial Action Coding System developed by Ekman and Friesen [17], and subjective participant ratings [3]. To minimize the influence of low-level perceptual factors on task performance, facial images were selected and modified for similarity on three primary bottom-up elements shown to influence attentional capture: color, form and luminance [32]. All facial images were greyscale on a black background, and all were similar in dimensions and in orientation. Further, all happy and angry faces displayed open-mouth expressions to minimize the chance that a featural difference in teeth exposure contributed to effects between the emotion types. To ensure that emotion types were matched on luminance, we used the Imaging Processing Toolbox in Matlab (Mathworks, Inc., Natick, MA) to quantify the luminosity (i.e., average pixel brightness intensity) of each image. Luminosity did not significantly differ between the anger, happy and neutral faces (*F* (2, 26) = .316, *p* = .732) or between any two emotions, using post hoc Tukey tests (Anger vs. Happy, *p* = .907; Anger vs. Neutral, *p* = .710; Happy vs. Neutral, *p* = .926). Thus, any subsequent difference found in behavioral and eye tracking performance between the emotion types is unlikely to be driven by emotion-related differences in color, form and luminosity.

For each trial, nine images were presented simultaneously in a 3×3 matrix measuring 26.8 cm (width) ×24.5 cm (height), and all matrices were viewed at a distance of approximately 70 cm with a visual angle of 20.9°×19.3°. Stimuli presentation and data collection were conducted on a 24-inch widescreen computer monitor with a resolution of 1024×768 pixels, using Tobii Studio software. A Tobii T60 XL eye tracker (Tobii Technology, Stockholm, Sweden), which uses the Pupil Center Corneal Reflection method to record the gaze of both eyes at a sampling rate of 60 Hz, with a spatial accuracy of approximately 0.5°, collected the eye tracking data.

### Design

To facilitate eye tracking acquisition and analysis, the original task was shortened from 162 to 81 matrices. Each matrix comprised nine different identities. One-third of the matrices (i.e., 27) were target-absent trials composed of only one type of emotional expression (i.e., nine trials each of matrices that were all neutral, all happy or all angry faces). The remaining two-thirds of the matrices (i.e., 54) were target-present trials composed of eight faces with the same emotional expression and one face with a discrepant emotional expression (e.g., one happy face among eight angry faces). Here, all combinations of categories (i.e., neutral-happy, neutral-angry, angry-happy, angry-neutral, happy-angry, happy-neutral) were utilized and consisted of nine trials each. Photo positions within the matrix were randomly assigned, such that target photos appeared in each position of the matrix only once. All 81 matrices were presented in a random order for each participant so that, on any given trial, participants did not know if a target would be present or, if a target was present, which it would be. RT and accuracy served as the dependent variables for replicating the face in the crowd effect.

### Procedure

Tested individually by a trained experimenter, participants were informed that they would see a series of matrices consisting of several faces with happy, angry, or neutral expressions and that their job was to press the “S” key, if all faces showed the same expression, and the “L” key, if one face showed a discrepant expression. Before beginning the experimental task, participants completed 18 practice trials that consisted of schematic faces as stimuli. These trials were used to familiarize participants with task design and stimulus timing. Between the practice trials and the experimental task, participant eye gaze was individually calibrated using a standard nine-point calibration. When the eye tracking software indicated that one or more points were not optimally acquired, the procedure was repeated until all points were calibrated successfully.

To ensure that all visual scanning patterns originated from the same location, each trial began with the presentation of a white fixation cross that was displayed in the middle of the screen for 500 ms. Then, matrices were presented one at a time, remaining onscreen for 2000 ms. Participants were instructed to respond as quickly and accurately as possible; they were also informed that they should try to respond while the matrix was still onscreen but that responses could be made after it disappeared. After participants responded, the prompt “Ready?” appeared onscreen to indicate that the next trial could begin. When ready, participants pressed the space bar to start the next trial, allowing them to move through the task at their own pace. The task took approximately six minutes to complete. Feedback was not provided during either the practice trials or the experimental tasks.

### Data Reduction and Statistical Analysis

Prior to conducting eye tracking analyses, we first confirmed the presence of the face in the crowd effect by analyzing the behavioral data. We examined whether angry targets were more quickly and accurately identified than happy targets via separate 2 (target type: angry vs. happy) ×2 (distractor type: neutral vs. emotional) repeated measures ANOVAs, one for RT and one for accuracy. Significant effects were then followed-up using two-tailed paired *t* tests to assess the prediction that angry targets would be identified more quickly and accurately than would happy targets, in both the classical search asymmetry paradigm (i.e., angry target in happy distractors vs. happy target in angry distractors) and the constant distractor paradigm (i.e. angry target in neutral distractors vs. happy target in neutral distractors). Although comparing RT and accuracy between angry and happy targets is the traditional approach for examining the face in the crowd effect, we also directly compared the effects of angry and happy crowds by assessing trials comprising the “constant target” paradigm (i.e., neutral target in angry distractors vs. neutral target in happy distractors). Finding that neutral targets were more accurately and/or more quickly identified in happy crowds than in angry crowds would provide support for the distractor processing hypothesis (i.e., that happy distractors are processed more efficiently than are angry distractors), and eye tracking can be used to support this conclusion. Two-tailed paired *t* tests compared accuracy and RT for this constant target comparison. Only correct responses were included in the RT analysis.

Inspection of RT data revealed two participants with RTs ≥3 SD slower than the group mean, and analyses conducted for each individual condition revealed no other participant outliers. To determine whether the inclusion of these two participants meaningfully affected results, we first conducted the repeated measures ANOVA for RT with them included and compared the results for the same ANOVA with them excluded. Results for these two analyses did not differ. Thus, because their inclusion did not affect RT results, they were retained in the sample for eye tracking analysis.

To directly assess the perceptual processes supporting RT and accuracy differences, eye tracking analyses were then conducted for significant behavioral effects. Patterns of visual attention were examined via Tobii Studio software fixation analysis. Fixations were defined as periods of stable gaze during which eye movement velocity was under 0.42 pixels/ms, requiring that a pair of subsequent fixations be at least 35 pixels apart. Such velocity-based algorithms are easy to implement, run efficiently, and perform equally as well as dispersion-based algorithms do [33]. Three distinct measures of eye gaze served as dependent variables: (a) latency to target (i.e., time from onset of matrix display to first fixation on the target), (b) target orienting (i.e., number of distractors fixated before the first fixation on the target), and (c) distractor processing (i.e., average duration of fixation time per distractor viewed before the first fixation on the target). The first of these outcome measures was used to determine if eye tracking output was consistent with behavioral findings, whereas the other two outcome measures examined whether the face in the crowd effect elicited via the current task is explained by differential attention-orienting to the target face and/or by differential processing efficiency of the distractor faces.

To affirm the validity of supplementing the traditional behavioral indices with the current eye tracking indices, a Pearson's *r* correlation coefficient was computed to assess the relationship between the latency to fixate targets and RT. A strong, positive correlation between latency to target and RT would provide further support that RT is influenced by how quickly the target is first fixated. A 2 (target type: angry vs. happy) ×2 (distractor type: neutral vs. emotional) repeated measures ANOVA was then conducted for latency to first target fixation. Follow-up two-tailed paired *t* tests assessed latency within both search paradigms.

Next, to examine the target orienting hypothesis, we conducted a 2 (target type: angry vs. happy) ×2 (distractor type: neutral vs. emotional) repeated measures ANOVA for number of distractor faces fixated before first target fixation. Follow-up two-tailed paired *t* tests assessed target orienting within both search paradigms. Finally, to examine the distractor processing hypothesis, we conducted a 2 (target type: angry vs. happy) ×2 (distractor type: neutral vs. emotional) repeated measures ANOVA for average duration of fixation time per distractor viewed before first fixation on the target. Follow-up, two-tailed paired *t* tests assessed distractor processing within both search paradigms. Two-tailed tests were used for all eye tracking follow-up tests because we did not explicitly predict confirmation of either the target orienting hypothesis or the distractor processing hypothesis.

Only correct trials in which the target was fixated before a participant responded were included in latency to target, target orienting, and distractor processing analyses. Doing so eliminated any extraneous eye gaze that occurred between a participant response and the end of the 2000 ms stimulus display. Furthermore, because the interstimulus fixation cross was presented in the center of the screen and subsequently biased first fixations to the middle position of the matrix, trials in which the target was in the middle position of the matrix (i.e., six of the total eighty-one matrices) were excluded from eye tracking analyses, reducing the target-present trials from nine to eight for each target-distractor combination. In such trials, targets were the first face fixated approximately 95% of the time and were identified more quickly and accurately. These six trials were therefore excluded from analyses because they revealed little about purposeful attention and were disproportionately represented in the correct trials of certain image types.

## Results

### Behavioral Performance

The repeated measures ANOVA for RT revealed a main effect of target type: consistent with the face in the crowd effect, angry targets were identified more quickly (1530 ms) than were happy targets (1642 ms; *F* (1, 32) = 15.41, *p*<.001, η_p_^2^ = .33). A main effect of distractor type also emerged, as emotional targets were found more quickly among neutral distractors (1494 ms) than among emotional distractors (1677 ms; *F* (1, 32) = 22.27, *p*<.001, η_p_^2^ = .41). The interaction between target and distractor type was also significant, demonstrating that the effect of distractor type was more pronounced when happy faces were targets, *F* (1, 32) = 4.88, *p* = .034, η_p_^2^ = .13. Post hoc examination revealed that, although angry targets were found more quickly in neutral crowds (1468 ms) than in happy crowds (1591 ms; *t* (32) = 3.62, *p* = .001, *d* = 0.28), this effect was even larger for happy target faces (1520 ms in neutral crowds and 1764 ms in angry crowds; *t* (32) = 4.21, *p*<.001, *d* = 0.51).

The repeated measures ANOVA for accuracy, like that for RT, revealed a main effect of target type: angry targets were identified more accurately (70.9%) than were happy targets (60.9%; *F* (1, 32) = 8.73, *p* = .006, η_p_^2^ = .21). Also similar to the RT analyses, a main effect of distractor type emerged, such that participants identified emotional targets more accurately in a crowd of neutral distractors (78.4%) than in a crowd of emotional distractors (53.4%; *F* (1, 32) = 55.47, *p*<.001, η_p_^2^ = .63). The interaction between target type and distractor type for accuracy was not significant, *F* (1, 32) = 1.39, *p* = .247, η_p_^2^ = .04, suggesting that angry targets were more accurately identified than happy targets similarly across distractor type.

**Figure 1:**
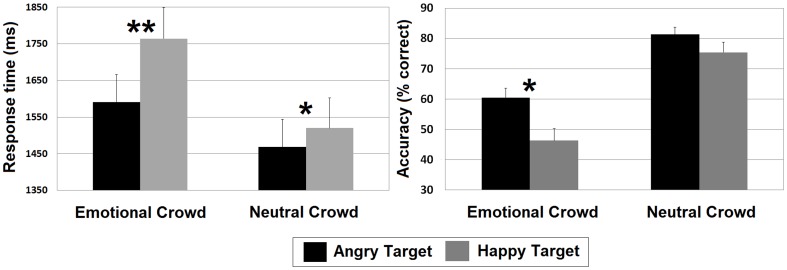
RT and accuracy. Mean response time (left) and accuracy (right) for correct responses on target-present trials. Vertical bars indicate SE. *Significant at p<.05. **Significant at p<.01.

Follow-up tests directly comparing RT and accuracy in the search asymmetry paradigm indicated that angry targets in happy crowds were identified more accurately (60.5%) and more quickly (1591 ms) than happy targets in angry crowds (46.3%, 1764 ms; *t* (32) = 2.39, *p* = .023, *d* = 0.70 for accuracy and *t* (32) = 3.46, *p* = .002, *d* = 0.37 for RT). Likewise, follow-up tests directly comparing RT and accuracy in the constant distractor paradigm revealed that angry targets in neutral crowds were found more quickly (1468 ms) than were happy targets in neutral crowds (1520 ms; *t* (32) = 2.05, *p* = .048, *d* = 0.11). The comparison on accuracy was in the expected direction but did not reach statistical significance (angry targets: 81.3%, happy targets: 75.4%; *t* (32) = 1.65, *p* = .108, *d* = 0.35). RT and accuracy differences are displayed in Figure 1.

Finally, an additional paired *t* test was conducted for the “constant target” paradigm to assess if neutral targets were identified more quickly and accurately in either happy or angry crowds. There was no significant difference for both RT (*t* (31) = 0.92, *p* = .365, *d* = 0.10) and accuracy (*t* (32) = 1.50, *p* = .143, *d* = 0.32), suggesting that angry and happy distractors did not affect behavioral responses for identifying neutral targets. Thus, eye tracking indices were not pursued for this comparison.

### Eye Tracking

**Figure 2:**
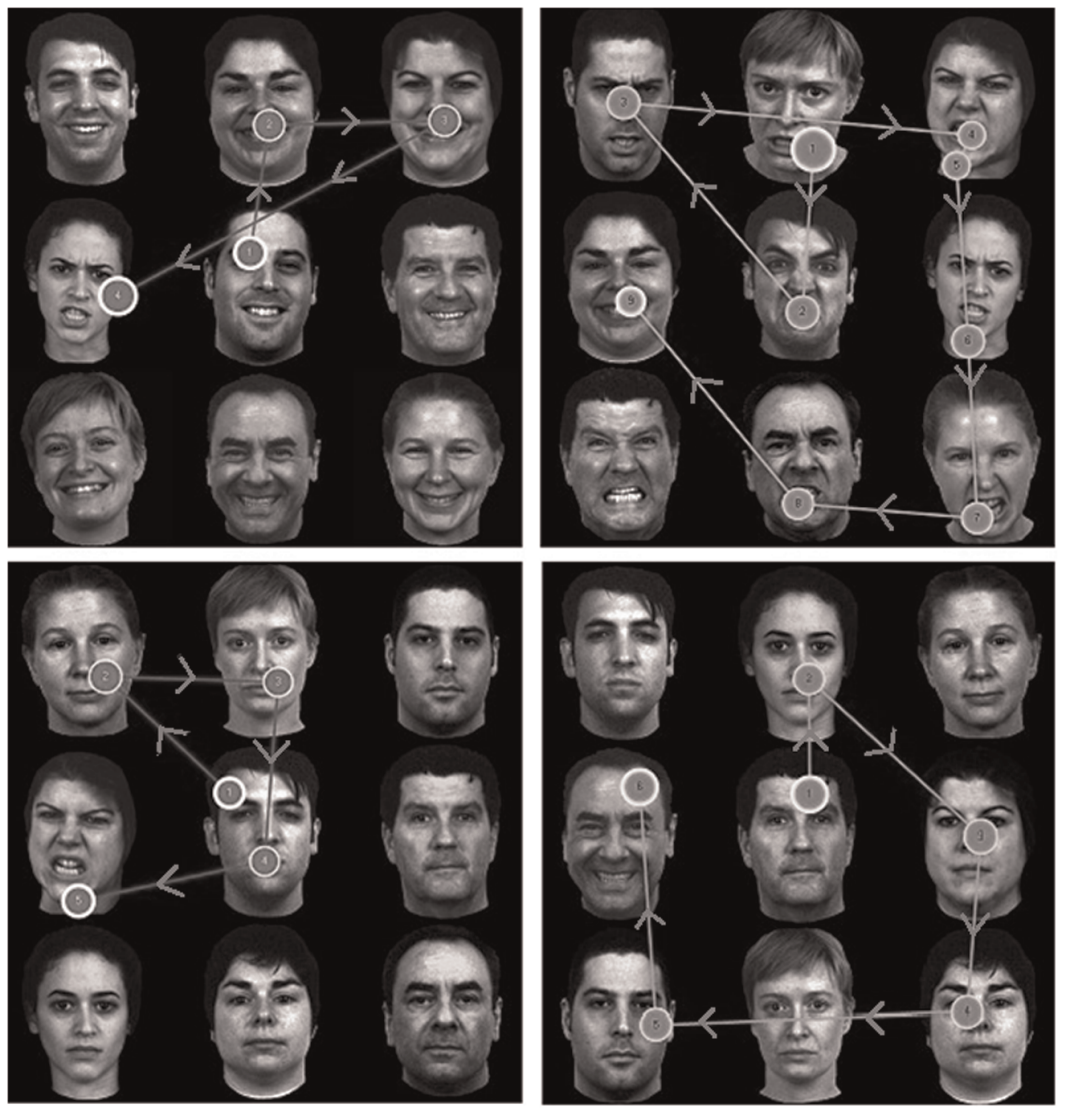
Example stimuli with superimposed fixation plots. Representative ordered fixation plots superimposed on sample stimulus matrices. Clockwise from the top left: an angry target as the fourth face fixated among happy distractors, a happy target as the eighth face fixated among angry distractors, a happy target as the sixth face fixated among neutral distractors, and an angry target as the fourth face fixated among neutral distractors. For demonstration purposes, all targets are in the left column and middle row. Circles represent fixation points; arrows denote directions of gaze paths.

Examples of eye tracking results superimposed on stimulus matrices are presented in Figure 2.

Latency to first fixate targets was examined to assess consistency with the behavioral results. There was a significant correlation between latency to target and RT (*r* (31) = .61, *p*<.001), confirming that gaze patterns related to behavioral patterns of RT. The repeated measures ANOVA for latency revealed a main effect of target type: consistent with the face in the crowd effect, participants registered their first fixation on angry targets more quickly (801 ms) than on happy targets (903 ms; *F* (1, 27) = 7.89, *p* = .009, η_p_^2^ = .23). There was also a main effect of distractor type, as participants first fixated targets more quickly in neutral crowds (810 ms) than in emotional crowds (894; *F* (1, 27) = 4.19, *p* = .051, η_p_^2^ = .13). The significant interaction between target type and distractor type demonstrated that the effect of distractor type was more pronounced when happy faces were targets, *F* (1, 27) = 5.41, *p* = .028, η_p_^2^ = .17. More specifically, post hoc examination revealed that latency to first fixate angry targets did not differ between happy crowds (805 ms) and neutral crowds (807 ms; *t* (32) = 0.04, *p* = .966, *d* = 0.00), but latency to first fixate happy targets was slower in angry crowds (995 ms) than in neutral crowds (810 ms; *t* (32) = 2.67, *p* = .013, *d* = 0.64).

**Figure 3:**
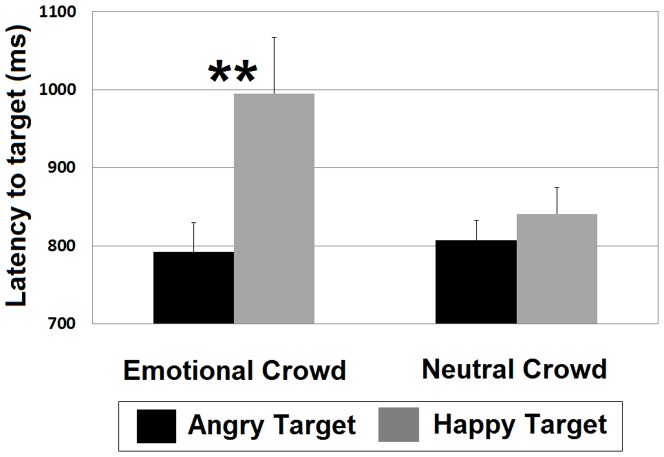
Latency to fixate target. Latency to target (mean time from stimulus display to first target fixation) on correct trials. *Significant at p<.05. **Significant at p<.01.

Follow-up tests directly comparing latency within the search asymmetry paradigm revealed that angry targets among happy crowds were first fixated more quickly (792 ms) than were happy targets among angry crowds (995 ms; *t* (27) = 2.75, *p* = .010, *d* = 0.64). In the constant distractor paradigm, latency to angry targets among neutral crowds (807 ms) did not significantly differ from latency to happy targets among neutral crowds (841 ms; *t* (32) = 0.97, *p* = .341, *d* = 0.20). Latency results can be viewed in Figure 3.

**Figure 4:**
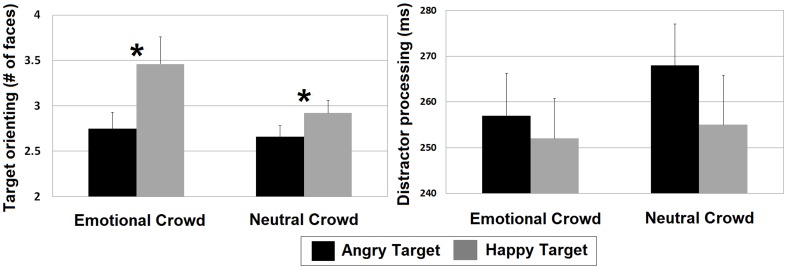
Target orienting and distractor processing. Target orienting (average number of distractors fixated before first target fixation) on the left and distractor processing (average duration of fixation time per distractor viewed before first target fixation) on the right for correct trials. *Significant at p<.05.

The repeated measures ANOVA for the target orienting hypothesis revealed a significant main effect of target type: participants fixated fewer distractors before first fixating angry targets (2.71) than before first fixating happy targets (3.20; *F* (1, 27) = 14.80, *p* = .001, η_p_^2^ = .35). There was no main effect of distractor type, *F* (1, 27) = 2.86, *p* = .102, η_p_^2^ = .10, indicating that the number of neutral and emotional distractors fixated prior to target did not significantly differ. The interaction between target type and distractor type was also not significant, *F* (1, 27) = 1.56, *p* = .223, η_p_^2^ = .06, suggesting that, regardless of distractor type, fewer distractors were fixated before first fixating angry targets than before first fixating happy targets. To determine if the target orienting advantage for angry faces persisted across both the search asymmetry and constant distractor paradigms, follow-up tests directly assessed the target orienting hypothesis within each. For the search asymmetry paradigm, fewer happy distractors were fixated before first fixating angry targets (2.75) than angry distractors were fixated before first fixating happy targets (3.46; *t* (27) = 2.51, *p* = .018, *d* = 0.53). Similarly, within the constant distractor paradigm, fewer neutral distractors were fixated before first fixating angry targets (2.66) than before first fixating happy targets (2.92; *t* (32) = 2.07, *p* = .047, *d* = 0.35). These results suggest that the target orienting hypothesis is supported in the current task across both search paradigms (see Figure 4).

The repeated measures ANOVA for distractor processing revealed no significant main effect of target type (*F* (1, 27) = 1.33, *p* = .259, η_p_^2^ = .05), no significant main effect of distractor type (*F* (1, 27) = 2.15, *p* = .154, η_p_^2^ = .07), and no significant interaction between target type and distractor type (*F* (1, 27) = 0.02, *p* = .885, η_p_^2^ = .00). Follow-up comparisons directly examining the search asymmetry paradigm also indicated no significant difference between the processing efficiency of angry distractors with happy targets (252 ms) and of happy distractors with angry targets (257 ms; *t* (27) = 0.51, *p* = .615, *d* = 0.10). Moreover, within the constant distractor paradigm, there was no significant difference between the processing efficiency of neutral distractors with angry targets (268 ms) and with happy targets (255 ms; *t* (32) = 1.53, *p* = .136, *d* = 0.21). Thus, we see no support that the face in the crowd effect elicited by the current task can be explained by processing efficiency of distractors in either the search asymmetry paradigm or the constant distractor paradigm (Figure 4).

## Discussion

The current study used eye tracking to directly assess the perceptual processes underlying performance on a face in the crowd task. Consistent with our behavioral data replicating the face in the crowd effect, participants registered their first visual fixation more quickly on angry targets than on happy targets. Eye tracking analyses were then used to evaluate two possible perceptual explanations of the effect: (1) the “target orienting” hypothesis (i.e., angry targets orient attention more quickly than do nonthreatening targets) and (2) the “distractor processing” hypothesis (i.e., nonthreatening distractors paired with an angry target are processed more efficiently than vice versa, leading to quicker detection of angry targets). Results revealed much stronger support for the target orienting hypothesis. Across both the search asymmetry and the constant distractor paradigms, fewer distractors were fixated before the first fixation on angry targets relative to happy targets. Eye tracking revealed no support for the distractor processing hypothesis, as no difference was found between the duration of fixation time on happy and angry distractors viewed before emotional targets. Furthermore, there were no accuracy or RT differences for trials of neutral targets paired with either angry or happy distractors. Taken together, these results indicate that angry target faces on the Face in the Crowd task initially attract or orient visual attention but do not disproportionately hold or “capture” attention in the traditional sense. If that were the case, longer fixations on angry distractors would have been evident.

Our findings of greater support for the target orienting hypothesis are consistent with studies showing that negative affect faces can increase preattentive guidance more so than can positive affect faces [20]. Angry faces may be ascribed enhanced environmental salience and, thus, disproportionately attract attention [2] [6]. Support for the target orienting hypothesis persisted across both the search asymmetry paradigm and the constant distractor paradigm, suggesting that the salience of angry targets was unaffected by the makeup of the crowd.

The lack of support for the distractor processing hypothesis is inconsistent with prior studies showing that happy faces are identified more quickly and accurately than are angry faces, when presented individually [22] [23] [24], and with those demonstrating delayed visual disengagement from angry faces relative to happy and neutral faces [25]. The apparent discrepancies between our findings and these studies are likely driven by differences in the processing of emotional stimuli that are individually presented compared to collectively presented. In this way, the findings reported here may reflect not only attention-orienting to angry faces but also unique perceptual processes involved in the simultaneous assessment of multiple salient stimuli [34] [35].

Although support for the target orienting hypothesis and lack of support for the distractor processing hypothesis extended across both the search asymmetry and constant distractor paradigms, in some respects the constant distractor paradigm produced less robust results. In neutral crowds, angry targets were identified significantly more quickly than were happy targets, but they were not identified more accurately, nor were they fixated first more rapidly (though both effects were in the direction that favored angry targets). These results align with prior research demonstrating that the face in the crowd effect is more pronounced when emotional distractors are used relative to when neutral distractors are used [7]. Although some have argued that weaker results in the constant distractor paradigm undermine the validity of the face in the crowd effect [7], an alternative explanation posits that neutral distractors are not the perfect perceptual intermediate between happy and angry faces and thus could enhance search efficiency for targets that, depending on the particular study, are more perceptually different from neutral distractors [1]. Further, finding an effect for RT but not for latency on the constant distractor paradigm raises the possibility that the presence of an all neutral crowd may enable participants to detect a discrepant emotional target peripherally (i.e., without registering a fixation on the target). RT and latency effects were more similar in the search asymmetry paradigm, where peripheral detection may be less advantageous given that here both the crowd and the target depict emotional valence, making detection of difference more difficult.

Importantly, however, we not only found that angry targets were identified more quickly than were happy targets in neutral crowds, but we also found that fewer neutral distractors were fixated in trials with angry targets relative to those with happy targets. Thus, because the current findings support the target orienting hypothesis even within neutral crowds, the face in the crowd effect cannot solely be driven by the effects of emotional distractors, which has been the main concern with search asymmetry designs [5] [6]. Additionally, higher accuracy rates and faster RTs in the constant distractor paradigm relative to the search asymmetry paradigm align with the very few studies that have implemented both [3]. Emotional targets may be found more quickly in neutral crowds than in emotional crowds because of the lack of affect and/or markedly salient perceptual features (e.g., displayed teeth) in neutral distractors. These features may have rendered them less salient than emotional distractors, resulting in more efficient search in the constant distractor paradigm. Such an explanation is also consistent with the finding that, overall, emotional distractors slowed target detection more so than neutral distractors. Although the effect was apparent for both types of emotional distractors, it was significantly larger for angry compared to happy distractors. This finding, however, is likely not indicative of a disproportionate distractor effect of angry faces in the search asymmetry paradigm, but rather a failure for happy targets to orient attention to the same degree as angry targets. Indeed, accuracy and RT for neutral targets did not differ between angry and happy distractors, suggesting that each type of emotional distractor produces similar effects on target detection.

The present study also provides further evidence that the face in the crowd effect likely reflects a real-world phenomenon. The Face in the Crowd task [3] used here produced results with veridical faces and a true crowd of multiple identities that are consistent with studies using dynamic stimuli [12] and those using well-controlled but less ecologically valid schematic stimuli [2] [6]. It should be noted, however, that several other studies using real faces have not detected an advantage for threatening faces [7] [8] [9] [10]. This discrepancy in results might arise from an emphasis in prior studies on maximizing perceptual similarity between each face by using the same identity in the crowd, cropping out salient features (e.g., the ears and hair), and/or manipulating the amount of teeth displayed on each face. Though such manipulation allows researchers to control for perceptual differences between facial expressions that may contribute to search advantages in these tasks, doing so may also result in homogeneous distractors that do not fully reflect a naturalistic crowd. Considering that emotionally expressive variations and low-level perceptual differences naturally occur between individuals and that the face in the crowd effect is rooted in evolutionary theory [1], stimuli with natural perceptual differences between faces may be important for future research.

Though the current study provides valuable insight into the perceptual processes underlying the face in the crowd effect, some limitations should be noted when interpreting these findings. First, to accommodate the demands of eye tracking analyses, the current study included fewer trials than did previous work that used the same task [3] and therefore may have had reduced power to detect behavioral effects. For example, unlike Pinkham and colleagues [3], we did not find an interaction between target type and distractor type for accuracy, suggesting no difference between the accurate detection of angry faces in the presence of happy versus neutral distractors. The failure to replicate this specific interaction, however, was not representative of replication generally: despite fewer trials, almost all behavioral effects that Pinkham and colleagues [3] found were also found in the present study, demonstrating that this task reliably elicits a search advantage for angry targets.

Second, although the emotional categories were matched on three primary low level perceptual properties, color, form and luminance [32], other perceptual elements (e.g., spatial frequency) may have differed. Demonstration of an ecologically valid face in the crowd effect requires the use of real facial stimuli and heterogeneous crowds, but this approach may sacrifice some control relative to the use of schematic faces. Future studies may therefore seek to specify the perceptual characteristics of real-world angry faces that support the face in the crowd effect. Rather than invalidating the effect, determination of these differences may illuminate the mechanisms by which angry faces orient attention. The intensity of expression was also not systematically controlled. Considering that the angry expressions used here (i.e., anger with teeth displayed) are less frequently encountered in common social interaction than are happy and neutral faces, future work should examine how emotional intensity and familiarity might affect results [36].

Third, despite relatively high levels of accuracy on the task as a whole (i.e. 73%), accuracy on the “happy targets in angry crowds” condition was at chance level, likely reflecting a difficulty for efficiently navigating angry crowds to detect another emotional category within the 2000 ms exposure time. Indeed, latency analyses demonstrate that targets were not fixated on nearly a third of trials in this condition, and participants were 50% more likely to select an incorrect “same” response than a correct “different” response when the target was never fixated. Thus, low accuracy rates on this condition may have been driven by participants adopting a “conservative criterion” when a target was not located, leading to a disproportionate number of “same” responses. Importantly, however, the primary analyses focusing on RT and eye tracking were constrained to correct trials to lessen the importance of accuracy rates. A post-hoc test on this condition demonstrated that RT for accurate trials (1763 ms) was significantly faster than RT for incorrect trials (1884 ms), *t* (31) = 2.05, *p* = .049, *d* = .24, confirming that chance level accuracy on this condition did not indicate indiscriminate responding or invalid RT data.

In closing, our results indicate that the face in the crowd effect elicited by the task used here is driven by the specific perceptual pattern of angry faces disproportionately orienting visual attention relative to other facial expressions. In contrast, we found no support for the hypothesis that the effect is driven by more efficient processing of distractor faces when angry faces are the target. Thus, these findings suggest that the advantage for detecting angry faces within the Face in the Crowd task [3] is likely to reflect enhanced salience of threatening stimuli and provides further support that this task is an ecologically valid and sensitive measure for eliciting the face in the crowd effect.

## References

[1] Horstmann G, Bauland A (2006) Search asymmetries with real faces: Testing the anger superiority effect. Emotion 6: 193–207. 16768552 10.1037/1528-3542.6.2.193

[2] Öhman A, Lundqvist D, Esteves F (2001) The face in the crowd revisited: A threat with schematic stimuli. J Pers Soc Psychol 80: 381–396. 11300573 10.1037/0022-3514.80.3.381

[3] Pinkham AE, Griffin M, Baron R, Sasson NJ, Gur RC (2010) The face in the crowd effect: Anger superiority when using real faces and multiple identities. Emotion 10: 141–146 10.1037/a0017387 20141311

[4] Fox E, Damjanovic L (2006) The eyes are sufficient to produce a threat superiority effect. Emotion 6: 534–539. 16938095 10.1037/1528-3542.6.3.534 PMC1852642

[5] Eastwood JD, Smilek D, Merikle PM (2001) Differential attentional guidance by unattended faces expressing positive and negative emotion. Percept Psychophys 63: 1004–1013. 11578045 10.3758/bf03194519

[6] Fox E, Lester V, Russo R, Bowles RJ, Pichler A, et al (2000) Facial expressions of emotion: Are angry faces detected more efficiently? Cogn Emot 14: 61–92. 17401453 10.1080/026999300378996 PMC1839771

[7] Juth P, Lundqvist D, Karlsson A, Öhman A (2005) Looking for foes and friends: Perceptual and emotional factors when finding a face in the crowd. Emotion 5: 379–395. 16366743 10.1037/1528-3542.5.4.379

[8] Damjanovic L, Roberson D, Athanasopoulos P, Kasai C, Dyson M (2010) Searching for Happiness Across Cultures. J Cogn Cult 10: 85–107 10.1163/156853710X497185

[9] Williams MA, Moss SA, Bradshaw JL, Mattingley JB (2005) Look at me, I'm smiling: Visual search for threatening and nonthreatening facial expressions. Vis Cogn 12: 29–50.

[10] Becker D, Anderson US, Mortensen CR, Neufeld SL, Neel R (2011) The face in the crowd effect unconfounded: Happy faces, not angry faces, are more efficiently detected in single- and multiple-target visual search tasks. J Exp Psychol Gen 140: 637–659 10.1037/a0024060 21744984

[11] Calvo MG, Nummenmaa L (2008) Detection of emotional faces: Salient physical features guide effective visual search. J Exp Psychol Gen 137: 471–494 10.1037/a0012771 18729711

[12] Ceccarini F, Caudek C (2013) Anger superiority effect: The importance of dynamic emotional facial expressions. Vis Cogn 21: 498–540 10.1080/13506285.2013.807901

[13] Frischen A, Eastwood JD, Smilek D (2008) Visual search for faces with emotional expressions. Psychol Bull 134: 662. 18729567 10.1037/0033-2909.134.5.662

[14] Horstmann G, Lipp OV, Becker SI (2012) Of toothy grins and angry snarls—Open mouth displays contribute to efficiency gains in search for emotional faces. J Vis 12: 1–15 10.1167/12.5.7 22637708

[15] Mermillod M, Vermeulen N, Lundqvist D, Niedenthal PM (2009) Neural computation as a tool to differentiate perceptual from emotional processes: The case of anger superiority effect. Cognition 110: 346–357 10.1016/j.cognition.2008.11.009 19128799

[16] Lundqvist D, Juth P, Öhman A (2013) Using facial emotional stimuli in visual search experiments: The arousal factor explains contradictory results. Cognition Emotion: In press. 10.1080/02699931.2013.867479 24341823

[17] Ekman P, Friesen WV (1978). *Manual of the Facial Action Coding System (FACS)*. Palo Alto, CA: Consulting Psychologists Press.

[18] Pinkham AE, Sasson NJ, Simpson CE, Healey K, Kohler C (2014) An intact threat superiority effect for non-social but not social stimuli in schizophrenia. J Abnorm Psychol: In press. 10.1037/a0035639 24661168

[19] Damjanovic L, Pinkham AE, Clarke P, Phillips J (2013) Enhanced threat detection in experienced riot police officers: Cognitive evidence from the face-in-the-crowd effect. Q J Exp Psychol: In press. 10.1080/17470218.2013.839724 24152089

[20] Reynolds MG, Eastwood JD, Partanen M, Frischen A, Smilek D (2008) Monitoring eye movements while searching for affective faces. Vis Cogn 17: 318–333 10.1080/13506280701623704

[21] Eastwood JD, Smilek D, Oakman JM, Farvolden P, van Ameringen M, et al (2005) Individuals with social phobia are biased to become aware of negative faces. Vis Cogn 12: 159–179.

[22] Sasson NJ, Pinkham AE, Richard J, Hughett P, Gur RE, et al (2010) Controlling for response biases clarifies sex and age differences in facial affect recognition. J Nonverbal Behav 34: 207–221.

[23] Elfenbein HA, Ambady N (2002) On the universality and cultural specificity of emotion recognition: A meta-analysis. Psychol Bull 128: 203–235. 11931516 10.1037/0033-2909.128.2.203

[24] Leppänen JM, Tenhunen M, Hietanen JK (2003) Faster choice-reaction times to positive than to negative facial expressions: The role of cognitive and motor processes. J Psychophysiol 17: 113–123.

[25] Belopolsky AV, Devue C, Theeuwes J (2011) Angry faces hold the eyes. Vis Cogn 19: 27–36 10.1080/13506285.2010.536186

[26] Hampton C, Purcell DG, Bersine L, Hansen CH (1989) Probing ‘pop-out’: Another look at the face-in-the-crowd effect. Bull Psychon Soc 27: 563–566.

[27] Purcell DG, Stewart AL (2010) Still another confounded face in the crowd. Atten Percept Psychophys 72: 2115–2127 10.3758/APP.72.8.2115 21097856

[28] Findlay JM (1997) Saccade target selection during visual search. Vision Res 37: 617–631. 9156206 10.1016/s0042-6989(96)00218-0

[29] Williams DE, Reingold EM, Moscovitch M, Behrmann M (1997) Patterns of eye movements during parallel and serial visual search tasks. Can J Exp Psychol 51: 151–164. 9340075 10.1037/1196-1961.51.2.151

[30] Zelinsky GJ, Sheinberg DL (1997) Eye movements during parallel-serial visual search. J Exp Psychol Hum Percept Perform 23: 244–262. 9090154 10.1037//0096-1523.23.1.244

[31] Gur RC, Sara R, Hagendoorn M, Marom O, Hughett P, et al (2002) A method for obtaining 3-dimensional facial expressions and its standardization for use in neurocognitive studies. J Neurosci Meth 115: 137–143. 10.1016/s0165-0270(02)00006-7 11992665

[32] Turatto M, Galfano G (2000) Color, form and luminance capture attention in visual search. Vision Res 40: 1639–1643. 10814751 10.1016/s0042-6989(00)00061-4

[33] Salvucci DD, Goldberg JH (2000) Identifying fixations and saccades in eye-tracking protocols. In: Proceedings of the Eye Tracking Research and Applications Symposium. New York: ACM Press. pp. 71–78.

[34] Hariri AR, Bookheimer SY, Mazziotta JC (2000) Modulating emotional responses: Effects of a neocortical network on the limbic system. Neuroreport 11: 43–48. 10683827 10.1097/00001756-200001170-00009

[35] Öhman A, Mineka S (2001) Fears, phobias, and preparedness: Toward an evolved module of fear and fear learning. Psychol Rev 108: 483–522. 11488376 10.1037/0033-295x.108.3.483

[36] Shen J, Reingold EM (2001) Visual search asymmetry: The influence of stimulus familiarity and low-level features. Percept Psychophys 63: 464–475. 11414134 10.3758/bf03194413

